# Hypodiploidy in a pediatric patient of T-cell acute lymphoblastic leukemia: a case report

**DOI:** 10.1186/s12920-021-01023-9

**Published:** 2021-07-03

**Authors:** Martyna Stefaniak, Gabriela Ręka, Joanna Zawitkowska, Monika Lejman

**Affiliations:** 1 Student Scientific Society, Laboratory of Genetic Diagnostics, Medical University of Lublin, A. Gębali 6, 20-093 Lublin, Poland; 2 Department of Paediatric Haematology and Oncology and Transplantology, Medical University of Lublin, A. Gębali 6, 20-093 Lublin, Poland; 3 Laboratory of Genetic Diagnostics, Medical University of Lublin, A. Gębali 6, 20-093 Lublin, Poland

**Keywords:** Acute lymphoblastic leukemia, Hypodiploidy, FISH, Microarray

## Abstract

**Background:**

T-cell acute lymphoblastic leukemia is a subtype of acute lymphoblastic leukemia, one of the most common childhood neoplasms. Hypodiploidy is a chromosome abnormality with fewer than 45 chromosomes and is associated with unsatisfactory clinical outcomes in acute lymphoblastic leukemia.

**Case presentation:**

We report clinical and genetic findings of a 14-year-old male with T-cell acute lymphoblastic leukemia with low-hypodiploidy. The medical history included neck pain for a month, facial nerve palsy on the right side for 6 days, fever, drowsiness, and weakness for 3 days, vomiting, diarrhea for 1 day. The physical examination presented features of hypovolemia, palsy of the facial nerve on the right side, enlarged lymph nodes, hepatosplenomegaly, sore throat, and petechiae of the skin. Radiological images indicated lesions of different organs. Bone marrow biopsy confirmed precursor T-ALL. In the FISH tests, *KMT2A* and *BCR/ABL1* rearrangements were not observed. GTG banding revealed 3 cell clones, which confirmed the hypodiploidy. Multiplex RT-qPCR was performed. *STIL/TAL1* (del1p32) gene rearrangement was found in the blast cells. Additional tests were performed using the CytoScan HD microarray technique. Molecular karyotype did not reveal hypodiploidy, but identified other abnormalities such as duplication of chromosomal regions: 4q25q35.2, 6p23.3p11.1 and 8p23.3q24.21, and the loss of heterozygosity of short arm chromosome 9. In two regions of the chromosome biallelic deletions were found at 9p21.3, including the *CDKN2A*, *CDKN2B, IFNA1, MTAP* genes and at 10q23.31, containing *PTEN.* The child died 9 days after diagnosis.

**Conclusions:**

Bone marrow biopsy, GTG banding, FISH techniques, and molecular karyotyping were used to make an accurate diagnosis. This case documents a rapid progression of the disease and unfavorable results of T-cell acute lymphoblastic leukemia with hypodiploidy.

## Background

T-cell acute lymphoblastic leukemia accounts for 15% of total childhood cases of ALL [1]. Hypodiploid ALL can be divided into cytogenetic subtypes: near-haploidy (23–29 chromosomes), low-hypodiploidy (30–39 chromosomes), high-hypodiploidy (40–43 chromosomes), and high hypodiploidy with 44 chromosomes [2–4]. Hypodiploidy is present in 5–8% of ALL cases of the general population [5]. The 5-year event-free survival rates in T-ALL have been constantly improving due to recent advancements in therapy and they are now exceeding 85% in many latest clinical trials. However, patients with hypodiploid ALL have continued to fare poorly [6–9]. There are cases when hypodiploidy is “masked” with no karyotypically apparent clone with ≤ 43 chromosomes, but their abnormal karyotypes contain 50–78 or even more chromosomes from duplicated previously hypodiploid cells [10].

We report clinical and genetic findings of a child with T-cell acute lymphoblastic leukemia with low-hypodiploidy.

## Case presentation

Clinical features: A 14-year-old male presenting with hyperleukocytosis and thrombocytopenia was admitted to the Department of Paediatric Haematology and Oncology and Transplantology on 30 June, 2019. The medical history included: neck pain for a month, facial nerve palsy on the right side for 6 days, fever, drowsiness, and weakness for 3 days, vomiting, diarrhea for 1 day. Family history was negative, and the patient did not suffer from coexisting diseases. The physical examination presented the following: features of hypovolemia, palsy of the facial nerve on the right side, enlarged bilateral cervical, axillary, inguinal, and left supraclavicular lymph nodes (with a diameter of 1–1.5 cm), hepatosplenomegaly (liver protruding 2–5–6 cm from below the costal arch, spleen—3–4 cm), sore throat, petechiae of the skin. The ultrasound results were as follows: thyroid gland—a focal hypoechoic and heterogeneous lesion; testicles—an irregular, slightly hypoechoic area about 12 × 12 mm in the left testicle; the abdomen—hepatosplenomegaly, a horseshoe kidney with two enlarged lymph nodes above connection. The chest radiograph showed lymph nodes of the upper mediastinum. Magnetic resonance of the central nervous system presented numerous hemorrhagic areas in both cerebral hemispheres. In the echocardiography the left ventricular hypertrophy was described. Bone marrow biopsy with immunophenotyping confirmed precursor T-ALL (CD3 87%, CD5 89%, CD7 78%, CD10 4%, CD19 4%, CD20 1%, CD22 5%, CD 34 42%, HLA-DR 7%, CD11B 14%, CD13 16%, CD14 16%, CD15 6%, CD 33 13%, CD45 98%, CD117 0.3%, cyCD3 37%, cyCD22 2%, cyCD64 3%, cyCD79a 7%, cytoplasmic IgM 4%, myeloperoxidase 5%, terminal deoxynucleotidyl transferase 4%, kappa 1%, lambda 1%, surface IgM 1%). In the myelogram, 91.2% of the nucleated elements of the bone marrow were immature blast cells.

Due to increasing leukocytosis (624,130/µl), the patient was qualified for leukapheresis. Laboratory test results are shown in Table 1. The Arrow central venous catheter was inserted under general anesthesia on 2 July. After this procedure, the patient's condition worsened. The patient did not regain consciousness. There was bleeding from the oral mucosa. Tensions and tonic convulsions of the whole body were reported. Diazepam was applied with effect. Leukapheresis was performed, and leukocytes decreased to 451,140/µl. However, the patient’s condition deteriorated. Tachypnea (25 per minute), desaturation (80%), low blood pressure, and tachycardia (180 per minute) were observed and the patient was transferred to the Intensive Care Unit. Unfortunately, the child died 9 days after diagnosis.

**Table 1:** Laboratory tests of the described patient

Parameter	1 July	2 July	3 July
Erythrocytes (/µl)	4.78 × 10^6^ (N^a^)	4.28 × 10^6^ (N)	4.16 × 10^6^ (N)
Hemoglobin (g/dl)	12.7 (L)	11.3 (L)	11.0 (L)
Hematocrit (%)	36.5 (L)	33.9 (L)	32.4 (L)
Leukocytes (/µl)	417,000 (H)	624,130 (H)	451,140 (H)
Neutrophils (/µl)	0 (L)	0 (L)	0 (L)
Lymphocytes (/µl)	284,690	451,680 (H)	357,770 (H)
Thrombocytes (/µl)	65,000 (L)	71,000 (L)	74,000 (L)
Blasts (%)	88 (H)	–	–
Phosphates–inorganic phosphorus (mmol/l)	0.38 (L)	0.54 (L)	0.58 (L)
Creatinine (mg/dl)	1.33 (H)	1.33 (H)	1.64 (H)
Urea (mg/dl)	17.00 (L)	22.90 (N)	57.30 (H)
Uric acid (mg/dl)	16.25 (H)	0.03 (L)	1.34 (L)
C-reactive protein (mg/dl)	1.52 (H)	–	1.69 (H)
Creatine kinase-MB (U/l)	–	–	26.60 (H)
Troponin I (ng/ml)	–	–	6.07 (H)
NT-proBNP (pg/ml)	–	–	12,521 (H)
Lactate dehydrogenase (U/l)	12,735 (H)	–	6328 (H)

^a^*L* low, *N* normal, *H* high

Genetic tests: A 24-h non-stimulated culture revealed the somatic karyotype of the cells. GTG banding and FISH techniques were performed. In the FISH tests using *BCR/ABL1*, *KMT2A* molecular probes (Vysis, Abbott Molecular) no rearrangements were observed. GTG banding revealed 3 cell clones: 47<2n>,XY,t(4;8)(q23;q24.2),+der(8)t(4;8)(q25;q24),der(18)t(6;18)(p11;q22)[10]/30,Y,-X,-2,-3,-4,-5,der(8)t(4;8)(q25;q24),-10,-11,-12,-14,-16,-17,der(18)t(6;18)(p11;q22),-19,-20,-20,-21,-22[4]/35,XY,-3,-4,der(8)t(4;8)(q25;q24),-10,-11,-12,-16,-17,der(18)t(6;18)(p11;q22),-20,-21,-22[5], which confirmed the hypodiploid clone and complex karyotype.

**Fig. 1:**
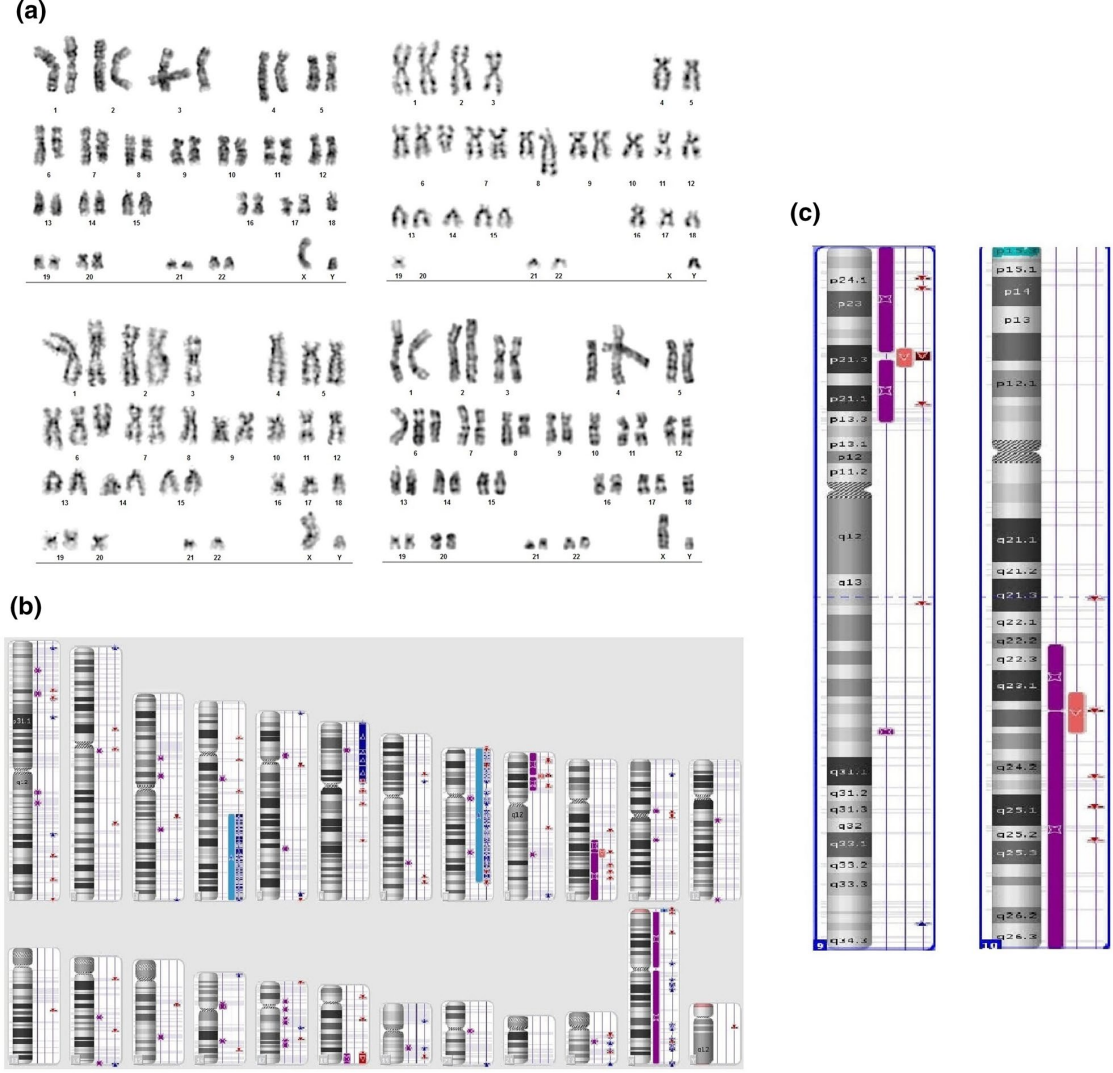
**a** Cytogenetic analysis of bone marrow cells at diagnosis. **b** Karyoview from microarray test and a scheme presenting chromosomal aberrations in the patient: duplication of 4q25q35.2, 6p23.3p11.1 and 8p23.3q24.21 chromosomal regions, deletions at 9p21.3 and 10q23.31, and loss of heterozygosity of the short arm of chromosome 9. Asterisks correspond to deletion (red colour), duplication (blue colour), and loss of heterozygosity (purple colour). **c** Karyoview from microarray test: chromosomes 9 and 10

Moreover, multiplex RT-qPCR was performed (Hemavision-28Q DNA Diagnostic). *STIL/TAL1* (del1p32) gene rearrangement was found in the blast cells. Additional tests were performed using the CytoScan HD microarray technique (Applied Biosystems, Thermo Fisher Scientific, Waltham, MA, USA) (Fig. 1a–c). The result was as follows: arr[GrCh37]4q25q35.2[109129049_190957473]x2~3,6p23.3p11.1[378555_58758772]x3,8p23.3q24.21[158048_1287705840x2~3,9p24.3p13.3(192128_35533348)x2 hmz,10q22.2q26.3(77126173_135426536)x2 hmz,18q22.2q23(68033599_78014123)x1 hmz.

The molecular karyotype did not reveal hypodiploidy, but showed other abnormalities such as duplication of chromosomal regions: 4q25q35.2, 6p23.3p11.1 and 8p23.3q24.21, and the loss of heterozygosity on the short arm of chromosome 9. In two regions of the chromosome biallelic deletions were found at 9p21.3 including the *CDKN2A*, *CDKN2B, IFNA1, MTAP* genes and at 10q23.31 containing *PTEN.* SNP array indicated monoallelic deletion in *STIL* (5′12 exon-end).

## Discussion and conclusions

In our study we report a rare case of a patient with childhood T-ALL and hypodiploidy. We consider that our case report is valuable because there is limited data in the literature concerning this subject. Despite the fact that hypodiploidy has been known for many years, there is little understanding of genomic mutations, the factors causing aneuploidy, or the biological basis of the illness’s unfavorable outcomes [8]. The latest mapping of additional genetic changes revealed that near- and low-hypodiploid ALL present unique mutational patterns and are dissimilar subtypes [2]. It was found that cases with 24–31 chromosomes contain changes targeting receptor tyrosine kinase- and Ras signaling (71%) and the IKZFa3 lymphoid transcription factor (AIOLOS; 13%). In comparison, low hypodiploid ALLs with 32–39 chromosomes are associated with older age, low presenting leukocyte count and TP53 mutations (91.2%) in germline cells, as well as alterations of RB1 (41%) and IKZF2 (HELIOS; 53%) [7].

Near haploidy and low hypodiploidy are both found in approximately 0.5% of acute lymphoblastic leukemia in children. In adults, 3 to 4% of ALL cases are low hypodiploidy or near triploid, while there are no records of near haploidy at that age. Recent studies have shown that there is equal frequency in males and females. The age profile of patients with near-haploid ALL is restricted to childhood with the median age of 5 years, while for low-hypodiploid ALL, which is more frequent with increasing age, the median is 11.5 years. In both groups there is a relatively low level of white blood cell counts at diagnosis (< 50 × 109/l), although the described patient had a high (417 × 109/l) level of leukocytes at diagnosis. T-cell ALL with 24–39 chromosomes as diagnosed in our proband is rarely reported in literature [2].

Patients affected with hypodiploid ALL are candidates for novel treatment regimens due to unsatisfactory clinical outcomes. Near-haploid and low-hypodiploid ALLs activate RAS and phosphatidylinositol 3-kinase (PI3K) signaling. On this basis a study on the sensitivity of cell lines and xenografts to MEK (functioning downstream of RAS), PI3K, and PI3K/mammalian target of rapamycin (mTOR) inhibitors was performed. The research revealed that PI3K and PI3K/mTOR inhibitors reduced proliferation in all investigated tumors. However, MEK inhibition did not have an effect. Consequently, inhibition of the PI3K pathway can possibly occur as a novel treatment option for hypodiploid ALL [2, 11]. Due to the unsatisfactory outcome of hypodiploid ALL with current treatment protocols, it’s necessary to see whether the newest targeted treatments may improve survival rate in these patients [2, 12]. Due to the deteriorating condition and sudden death of our patient, these treatment methods were not administered.

*TP53* alterations are connected with changes of the IKZF2 lymphoid transcription factor and the tumor-suppressor gene loci *CDKN2A* and *CDKN2B,* and can be found in almost all patients with low hypodiploidy in ALL. Low-hypodiploid ALL can be a manifestation of Li-Fraumeni syndrome, due to the fact that more than half the *TP53* mutations in low-hypodiploid ALL in children are found in non-tumor cells. Changes of *TP53* function might promote the typical aneuploidy characteristic of hypodiploid ALL, which enables us to understand the genetic pathogenesis of hypodiploid ALL [8]. Further genetic testing of family members of child patients with low-hypodiploid ALL and constitutional *TP53* mutations should be considered. There is a higher risk of future malignancies in survivors of low-hypodiploid ALL [2]. Due to the fact that the patient did not have chromosome 17 in the leukemic cells, the mutation in the *TP53* gene, which is common in low hypodiploidy in T-ALL children, was not tested [12].

In conclusion, abnormalities in the DNA of lymphoblasts help with further classification of the disease and are prognostic factors. Hypodiploidy in T-cell acute lymphoblastic leukemia is associated with unfavorable results, which was seen in the described case. GTG banding, FISH techniques, and CytoScan HD microarray techniques were used to make an accurate diagnosis.

## Data Availability

The datasets generated and/or analysed during the current study are available in the Gene Expression Omnibus (GEO) repository: https://www.ncbi.nlm.nih.gov/geo/query/acc.cgi?acc=GSE178261&fbclid=IwAR2FPjcxRKkttaKiBdJMvM4QxkBkp9v_Qe2M2nS6ZlPer8pJwyVHO_HXXys.

## Abbreviations

ALL: Acute lymphoblastic leukemia
T-ALL: T-cell acute lymphoblastic leukemia
FISH: Fluorescence in situ hybridization
GTG: Giemza banding
MRI: Magnetic resonance imaging
RT-qPCR: Real-time quantitative polymerase chain reaction
